# Motor Control: Correcting Errors and Learning from Mistakes

**DOI:** 10.1016/j.cub.2010.05.030

**Published:** 2010-06-25

**Authors:** Chris Miall

**Affiliations:** School of Psychology, University of Birmingham, Birmingham B15 2TT, UK

## Abstract

How do we learn from errors during complex movement tasks with redundancy? A new study shows that ambiguous mistakes in bimanual movements are corrected by the non-dominant hand, and responsibility for the error is assumed to fall to the effector with a recent history of poor performance.

## Main Text

In recent years, there have been a number of important theoretical developments which have revised the way we think about the control of human movement. Imagine a task such as playing a game of tennis. The challenges are daunting — the modern game is extraordinarily fast, and each action must be made at the limits of human reaction times, so that tennis strokes are planned and executed well before the ball arrives at the racquet. For a powerful player like Andy Roddick, the ball is served so fast — 155 mph — that it reaches the opponent's baseline in about 350 milliseconds. Estimating the ball's trajectory accurately enough to reach and return it requires the combination of incomplete sensory information from the visual system with prior knowledge of the ball's likely distribution of positions — Roddick tends to get the ball inside the tramlines more often than not. Kording and Wolpert [1] showed in a laboratory version of this task that we use a Bayesian approach in which we optimally integrate sensory information about the current event with prior knowledge of the distribution of past events.

Consider next the challenge for Roddick's opponent in returning the serve. He plans the swing of the racquet to reach the approaching ball, but again must integrate his motor plan with sensory feedback about its execution. The evolving act must be refined and modified as the latest sensory information is processed, specifying both where the ball is bouncing and how his race along the baseline is progressing. Todorov and Jordan [2] developed the theory of optimal feedback control, in which sensory feedback and prior knowledge are combined into a ‘state estimate’ of the current situation that is integrated with the goal of the action to dynamically specify the optimal motor responses required. The key concept is of ‘minimum intervention’. Control gains are adjusted according to the task, allowing irrelevant parameters to be uncontrolled (low gain) while task-critical parameters have high gain [3]. This was a significant advance over previous theories which could define an optimal plan in advance of an action [4–6], but could not easily modify the plan to deal with intrinsic variation in its execution or with changes in the external environment.

**Figure 1:**
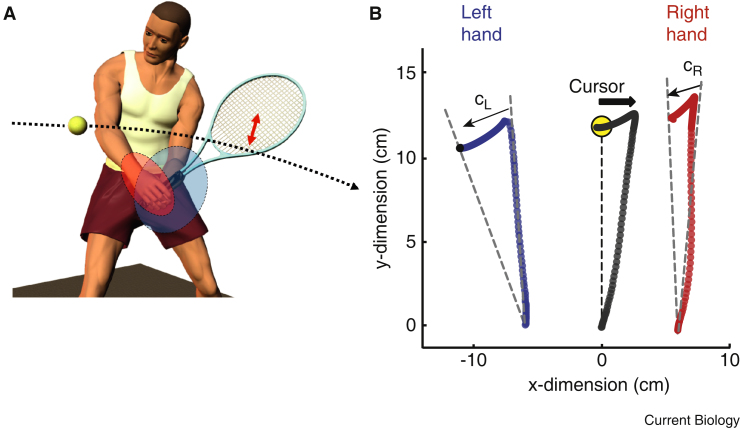
Assigning responsibility for motor errors. (A) Bimanual actions have redundancy because either or both arms can contribute to the action. So an error — missing the ball — could have been caused by a mistake from either arm. It might also be due to external events, such as a gust of wind. The ellipses indicate unequal certainty about the state of each arm. For right handers, the right arm is more reliable, less uncertain (red ellipse). So the mistake is more likely caused by the more uncertain left arm (blue). (B) Experimental design. The forward movement of a single cursor (centre) towards a target (yellow) is controlled by both unseen arms, but is rotated clockwise about its origin. The two hands share the correction (C_L_ and C_R_). On the subsequent trial, the two hands also adjust their initial direction to better control the rotated cursor. (Panel B adapted from [7].)

Now, take things a step further. Imagine when someone like me attempts to play tennis. I might be ambitious and try a two-handed backhand stroke. But it goes wrong (it always does!) and the racquet misses the ball (Figure 1A). Is it because my left arm was weak, or my right arm a bit slow? How should I untangle the ambiguity about the responsible effector muscles so that I can first correct the mistake, and second learn from the mistake to improve my performance? Because I am right-handed, my left arm is likely to be less accurate, so should I try to use my right arm to correct for the error, as it is better able to do so, or should I make my left arm correct the mistakes it was responsible for, and learn from them for next time?

As they report in this issue of *Current Biology*, White and Diedrichsen [7] have developed a clever experimental design to address these questions. Participants hold two lightweight mechanical arms and see the average of the two handle positions as a cursor on a screen which they must move towards a visual target (Figure 1B). Because the cursor reflects the average of both hands, the task is inherently redundant — one hand or the other could do all the work, or both hands can share the effort [8]. Likewise, an error — introduced experimentally by rotation of the path of the cursor around its start position — is ambiguous and could have been caused by either the left hand or the right hand misreaching. What the authors [7] first found is that for a group of right handers, the left hand corrected for the rotated cursor position more than the right hand. They confirmed this result by testing left handers, who are typically less lateralized than right handers, and found a weak effect in the opposite direction. Combing both groups, there was a strong relationship between handedness and the asymmetry of the corrections. The more dominant was one hand, the less likely it was to correct the error.

**Figure 2:**
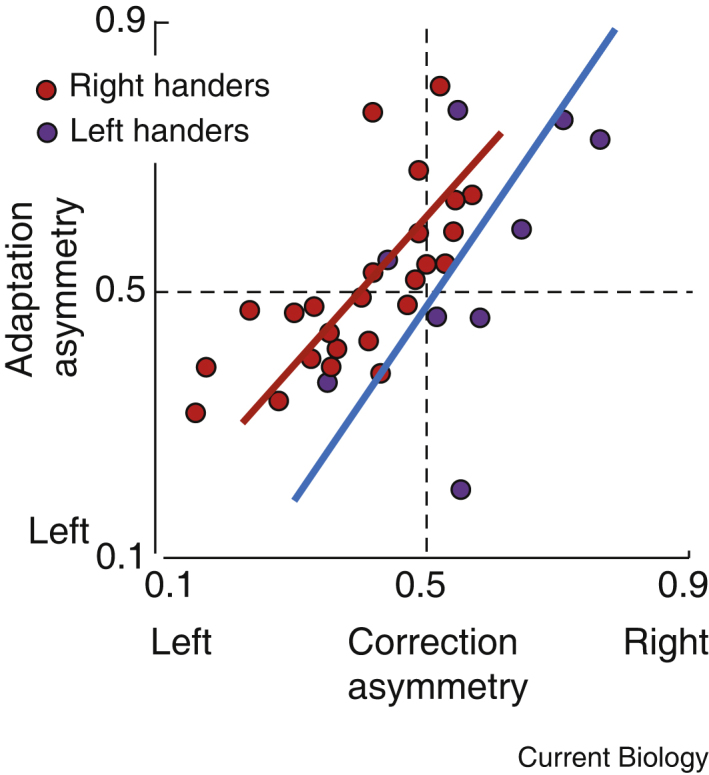
Learning to correct for motor errors. Across a group of participants, asymmetry of corrections was correlated to the asymmetry of subsequent adaptation, with the non-dominant hand correcting and adapting more. Follow-up experiments demonstrated that this effect was dependent on the recent history of errors — the hand making more errors learns more. (Adapted from [7].)

White and Diedrichsen [7] asked how the two hands adapted to these errors. Again, across the group there was a correlation between the asymmetry of error correction on one trial and the asymmetric shift in reaching direction on the next trial (Figure 2). The arm that corrected more learned more from the mistake. In an elegant twist, they then pre-exposed one hand or the other to a series of high errors, in a unimanual version of the task, before again testing the bimanual responses. Their results show that recent history of poor reaching performance is enough to bias the corrective responses and the learning towards the worse hand. We do force the bad arm to learn better, rather than rely on the good arm to do all the work.

Finally, White and Diedrichsen [7] addressed two alternative hypotheses to explain all these results. The motor system might bias responsibility for error to the arm for which it has less reliable information about performance. If predictions about the outcome of the dominant hand's action are better, because that hand is more reliable and more skilled, then ambiguous errors might be assigned to the less reliable, less predictable non-dominant hand (Figure 1A). And because prediction errors are an important training signal [9], the non-dominant hand would adapt more readily. Alternatively, the motor system might set the control gain higher for the less accurate hand, so that errors which are more likely to arise for that hand are more effectively corrected.

White and Diedrichsen [7] separated these two hypotheses with an experimental analogue of a gust of wind catching the tennis ball: the visual target, not the cursor, was suddenly shifted at movement onset. Now neither arm was responsible for the error, so inequality in the certainty of the two-state estimates should not result in asymmetric corrections, whereas inequality in control gains should. It turns out that the asymmetry of the responses does appear to be due to differences in control gain. This is a bit counter-intuitive, as much recent theory of motor control has shifted towards a more dominant role for prediction and state estimation [10–12]. However, the change in control gains is selective. Pre-training with target jumps did not affect later responses to cursor rotations, and *vice versa*.

This new paper [7] is neat, and may alter the way neuroscientists think about issues of generalization of skills from one hand to another [13]. It also opens some interesting new questions about neural representations in the motor system. We think the brain includes internal models that capture the response properties of the joints and muscles it controls [14], and probably has different models for different contexts — such as the behaviour of my arm with and without a tennis racquet in my hand. But do these models also code for the reliability of their internal estimates? Is the control gain set by this measure of reliability, so that a bad model has high gain, and must be pulled into line through a series of error corrections? Wolpert and Kawato [15] suggested that multiple internal models contribute to each motor command, combined according to their responsibility for control over the motor context. The model with high responsibility has more control. White and Diedrichsen's [7] results suggest an uncomfortable alternative: the models that are responsible for error, not for control, get the lion's share of the corrective task and of the learning that follows.

## References

[1] Kording K.P., Wolpert D.M. (2004). Bayesian integration in sensorimotor learning. Nature.

[2] Todorov E., Jordan M.I. (2002). Optimal feedback control as a theory of motor coordination. Nat. Neurosci..

[3] Scott S.H. (2004). Optimal feedback control and the neural basis of volitional motor control. Nature.

[4] Flash T., Hogan N. (1985). The coordination of arm movements: An experimentally confirmed mathematical model. J. Neurosci..

[5] Uno Y., Kawato M., Suzuki R. (1989). Formation and control of optimal trajectories in human multijoint arm movements: Minimum torque-change model. Biol. Cybern..

[6] Harris C.M., Wolpert D.M. (1998). Signal-dependent noise determines motor planning. Nature.

[7] White O., Diedrichsen J. (2010). Responsibility assignment in redundant systems. Curr. Biol..

[8] Guigon E., Baraduc P., Desmurget M. (2007). Computational motor control: redundancy and invariance. J. Neurophysiol..

[9] Tseng Y.W., Diedrichsen J., Krakauer J.W., Shadmehr R., Bastian A.J. (2007). Sensory prediction errors drive cerebellum-dependent adaptation of reaching. J. Neurophysiol..

[10] Molinari M., Restuccia D., Leggio M.G. (2009). State estimation, response prediction, and cerebellar sensory processing for behavioral control. Cerebellum.

[11] Miall R.C., Christensen L.O., Cain O., Stanley J. (2007). Disruption of state estimation in the human lateral cerebellum. PLoS Biol..

[12] Frens M.A., Donchin O. (2009). Forward models and state estimation in compensatory eye movements. Front. Cell Neurosci..

[13] Wang J., Sainburg R.L. (2007). The dominant and nondominant arms are specialized for stabilizing different features of task performance. Exp. Brain Res..

[14] Miall R.C., Wolpert D.M. (1996). Forward models for physiological motor control. Neural Netw..

[15] Wolpert D.M., Kawato M. (1998). Multiple paired forward and inverse models for motor control. Neural Netw..

